# Japanese Flounder HMGB1: A DAMP Molecule That Promotes Antimicrobial Immunity by Interacting with Immune Cells and Bacterial Pathogen

**DOI:** 10.3390/genes13091509

**Published:** 2022-08-23

**Authors:** Yuan Chen, Chao Yu, Shuai Jiang, Li Sun

**Affiliations:** 1 CAS and Shandong Province Key Laboratory of Experimental Marine Biology, Institute of Oceanology, Center for Ocean Mega-Science, Chinese Academy of Sciences, Qingdao 266071, China; 2 Laboratory for Marine Biology and Biotechnology, Pilot National Laboratory for Marine Science and Technology (Qingdao), Qingdao 266237, China; 3 College of Earth and Planetary Sciences, University of Chinese Academy of Sciences, Beijing 100049, China

**Keywords:** *Paralichthys olivaceus*, HMGB, DAMP, antimicrobial immunity

## Abstract

High mobility group box (HMGB) proteins are DNA-associated proteins that bind and modulate chromosome structures. In mammals, HMGB proteins can be released from the cell nucleus and serve as a damage-associated molecular pattern (DAMP) under stress conditions. In fish, the DAMP function of HMGB proteins in association with bacterial infection remains to be investigated. In this study, we examined the immunological functions of two HMGB members, HMGB1 and HMG20A, of Japanese flounder. HMGB1 and HMG20A were expressed in multiple tissues of the flounder. HMGB1 was released from peripheral blood leukocytes (PBLs) upon bacterial challenge in a temporal manner similar to that of lactate dehydrogenase release. Recombinant HMGB1 bound to PBLs and induced ROS production and the expression of inflammatory genes. HMGB1 as well as HMG20A also bound to various bacterial pathogens and caused bacterial agglutination. The bacteria-binding patterns of HMGB1 and HMG20A were similar, and the binding of HMGB1 competed with the binding of HMG20A but not vice versa. During bacterial infection, HMGB1 enhanced the immune response of PBLs and repressed bacterial invasion. Collectively, our results indicate that flounder HMGB1 plays an important role in antimicrobial immunity by acting both as a modulator of immune cells and as a pathogen-interacting DAMP.

## 1. Introduction

High mobility group box (HMGB) proteins belong to a family of DNA-binding factors that are located in the nucleus under normal physiological conditions. These proteins function in DNA replication, transcription, and repair [1]. The first HMGB was discovered in 1973 and was subsequently named “high mobility group” based on its high electrophoretic mobility in polyacrylamide gels [2,3]. To date, six HMGB family members have been identified, which are named HMGB1, HMGB2, HMGB3, HMGB4, HMG20A, and HMG20B [4,5].

HMGB1 is the most extensively studied HMGB family member. It is highly conserved in vertebrates and invertebrates [6]. The sequence of HMGB1 contains two tandem HMG boxes named box A and box B and a motif rich in acidic residues, e.g., aspartic acid and glutamic acid [7,8]. HMGB1 can be actively released during cytokine stimulation or passively when cells are lysed, and the released HMGB1 takes part in immunity by serving as a damage-associated molecular pattern (DAMP) [9,10,11,12,13]. Previous studies reported that the released HMGB1 can react with pattern recognition receptors such as the Toll-like receptors (TLRs) to trigger inflammatory responses [14,15,16,17]. In addition to interacting with host cells, HMGB1 can also function as a bactericidal agent [18,19,20]. Unlike HMGB1, HMG20A (also known as iBRAF) has only one HMG domain. HMG20A was reported to have important roles in developmental processes such as neuronal differentiation and maturation; in addition, the protein promotes the interstitial transformation of epidermal cells [21,22].

HMGB1 has been identified in a large number of teleosts, including zebrafish (*Danio rerio*) [23,24], red drum (*Sciaenops ocellatus*), grass carp (*Ctenopharyngodon idella*) [25], Japanese lamprey (*Lampetra japonica*), humphead snapper (*Lutjanus sanguineus*), pufferfish (*Tetraodon nigroviridis*), black rockfish (*Sebastes schlegelii*), goldfish (*Carassius auratus* L.), turbot (*Scophthalmus maximus*), and rohu (*Labeo rohita*) [26]. Some fish HMGB1 exhibit immunoregulatory effects. For example, the HMGB1 of goldfish, black rockfish, Japanese lamprey, and red drum can upregulate the expression levels of interleukin-6 (IL-6) and tumor necrosis factor-α (TNF-α) and promote macrophage proliferation [27,28]. However, much remains to be investigated with respect to the role of fish HMGB1 as a stress-induced DAMP in association with pathogen infection. Compared to HMGB1, the research on fish HMG20A is limited to Nile tilapia (*Oreochromis niloticus*), in which it was shown that HMG20A knockdown improved fish survival after bacterial infection [29].

Japanese flounder (*Paralichthys olivaceus*) is a fish species widely employed in aquaculture, especially in Asia. The genes of HMGB1 and HMG20A have been identified in flounder by genomic sequencing, but their functions have not been investigated. Herein, the expression and function of flounder HMGB1 as well as HMG20A were examined. We found that HMGB1 could be released from flounder immune cells upon bacterial challenge and that, in the extracellular milieu, HMGB1 exerted a significant effect on antibacterial immunity via direct interaction with both the host cells and the bacterial pathogens. Our findings provide new insights into the immunological function and regulation of fish HMGB proteins.

## 2. Materials and Methods

### 2.1. Animal Ethics

Japanese flounder were purchased from an aquaculture farm in Shandong Province, China. The fish were maintained in the laboratory and treated with tricaine methanesulfonate (Sigma, St. Louis, MO, USA) prior to tissue collection, as previously reported [30]. The experiments involving live animals were approved by the Ethics Committee of the Institute of Oceanology of the Chinese Academy of Sciences (permit No. MB2109-1).

### 2.2. Sequence Analysis

A sequence analysis was performed as described previously [31]. Briefly, the sequences of HMGB1 and HMG20A were analyzed using the simple modular architecture research tool (SMART) Version 7.0 [32]. A multiple sequence alignment of HMGB proteins was generated by DNAMAN.

### 2.3. Quantitative Real-Time Reverse Transcription-PCR (qRT-PCR)

qRT-PCR was performed as described previously [33]. The primers are listed in Table S1. The mRNA expression levels of HMGB1 and HMG20A were calculated using the comparative threshold cycle method (2^−ΔΔCT^), and β-actin was used as an internal reference [34].

### 2.4. Recombinant Protein Purification and Polyclonal Antibody Preparation

Recombinant HMGB1, HMG20A, and Trx were purified as previously described [31]. Briefly, the HMGB1 and HMG20A genes were amplified, and the PCR products were ligated into pET-28a (Novagen, San Diego, CA, USA) and transformed into *Escherichia coli* BL21 (DE3) competent cells. Recombinant Trx was expressed by pET-32a (Novagen, San Diego, CA, USA). The transformants were used to prepare the recombinant proteins with Ni-NTA agarose (QIAGEN, Dusseldorf, Germany). The purified proteins were washed with Triton X-114 to remove endotoxin. The protein purity was determined by SDS-PAGE and CBB R-250 staining (Figure S1). Antibodies against HMGB1 and HMG20A were prepared from immunized mice, as reported previously [33].

### 2.5. Detection of Extracellular HMGB1 and Lactate Dehydrogenase (LDH)

Flounder peripheral blood leukocytes (PBLs) were prepared by gradient centrifugation, as previously described [35]. The cells (1 × 10^7^ cells) were incubated with *Pseudomonas fluorescens* (1 × 10^7^ cells) for 4, 6, and 8 h; the supernatant was collected, and the extracellular proteins were prepared by trichloroacetic acid–acetone precipitation. To prepare whole-cell proteins, the cells were lysed using RIPA lysis buffer and then centrifuged at 15,000× *g* for 30 min at 4 °C. Immunoblotting was performed as previously described [36]. The primary antibodies were a mouse anti-HMGB1 antibody (1:1000 dilution) and a mouse anti-β-actin antibody (ABclonal, Wuhan, China) (1:3000 dilution); the secondary antibody was a horseradish peroxidase (HRP)-conjugated anti-mouse IgG antibody (ABclonal, Wuhan, China; 1:2000 dilution). To determine the release of LDH, flounder PBLs were incubated with *P. fluorescens,* as described above. LDH was measured with a CytoTox-ONE homogeneous membrane integrity assay kit (Promega, Leiden, The Netherlands). As a positive control, Triton X-100 (1%) was added to the cells to obtain the maximum LDH release.

### 2.6. Interaction of HMGB1 and HMG20A with PBLs

The protein binding to immune cells was analyzed by immunofluorescence microscopy, as previously reported [31]. Briefly, PBLs were plated in a cell culture dish (NEST, USA) for 1.5 h, followed by incubation with 5% skim milk for 1 h. After extensive washing with PBS, 50 μg/mL HMGB1, HMG20A, or Trx was added to the PBLs, and the cells were incubated at 22 °C for 2 h. The proteins that bound to PBLs were recognized by a mouse anti-His tag antibody and an Alexa Fluor Plus 488 labeled goat anti-mouse IgG antibody. The cells were fixed with 4% paraformaldehyde and incubated with 4, 6-diamino-2-phenylindole (DAPI) for 10 min. The images were taken with a confocal microscope (Carl Zeiss, Oberkochen, Germany).

### 2.7. Effects of HMGB1 and HMG20A on Immune Gene Expression, ROS Production, and Phagocytosis

Flounder PBLs (5 × 10^6^ cells/mL) were stimulated with HMGB1 (50 μg/mL), HMG20A (50 μg/mL), Trx (50 μg/mL), or PBS for 2, 6, or 12 h. The expression levels of proinflammatory cytokines, including IL-1β, IL-6, IL-10, TNF-α, and TGF-β1, were determined by qRT-PCR. ROS production was measured as previously described [37]. Briefly, PBLs were first incubated with DCFH-DA at a final concentration of 10 μM for 30 min, plated in 96-well plates (1 × 10^6^ cells/well), and incubated with HMGB1 (50 μg/mL), Trx (50 μg/mL), or PBS. After incubation for 2 h, ROS production was measured using a fluorescence spectrophotometer (Infinite M1000, Tecan, Switzerland). The phagocytosis of bacteria by PBLs was determined by flow cytometry, as previously reported [30].

### 2.8. Interaction of HMGB1 and HMG20A with Bacteria

*Vibrio harveyi*, *Vibrio anguillarum*, *Pseudomonas fluorescens*, *Edwardsiella tarda*, *Escherichia coli*, and *Streptococcus iniae* were cultured as reported previously [33]. The proteins that bound to bacteria were measured by ELISA [33]. Briefly, bacteria were coated on microtiter plates and blocked with 5% skim milk. HMGB1, HMG20A, or Trx were added to the plates at different concentrations, and the plates were incubated as described above at 22 °C for 2 h. The bound protein was recognized by the mouse anti-His tag antibody and the HRP-labeled goat anti-mouse IgG antibody. Colorimetric detection was performed by using a tetramethylbenzidine (TMB) kit. The binding index was defined as follows: *A*_450_ of HMGB1 or HMG20A/*A*_450_ of the PBS control. A microscopic examination of HMGB1 and HMG20A binding to bacteria was performed as reported previously [30]. To examine the competitive effect of HMGB1 on the binding of HMG20A to bacteria, *P. fluorescens* and *S. iniae* in coating buffer were added to 96-well microtiter plates. The plates were blocked as mentioned above. HMGB1 (1 mg/mL), Trx (1 mg/mL), or PBS were added to the plates and incubated at 22 °C for 2 h. After extensive washing, HMG20A (4 μg/mL) was added to the wells and incubated as described above. The competitive effect of HMG20A on the binding of HMGB1 to bacteria was determined similarly, except that the bacteria in the plates were pre-treated with HMG20A, followed by incubation with HMGB1. The bound HMGB1 or HMG20A was detected with the primary antibodies against HMGB1 or HMG20A and then with an HRP-labeled secondary antibody (1:2000 dilution).

### 2.9. Effects of HMGB1 and HMG20A on Bacterial Agglutination

*P. fluorescens* was resuspended in TBS to 10^9^ cells/mL with or without 10 mM CaCl_2_. The bacterial cells were then mixed with HMGB1 and HMG20A (100 μg/mL) and incubated at 22 °C for 2 h, followed by DAPI staining. Bacterial agglutination was observed with a confocal microscope (Carl Zeiss, Oberkochen, Germany).

### 2.10. Effects of HMGB1/HMG20A on Bacterial Infection and ROS Production in PBLs

To determine the effect of HMGB1/HMG20A on bacterial infection, *P. fluorescens* was pre-treated with HMGB1 (50 μg/mL), HMG20A (50 μg/mL), Trx (50 μg/mL), or PBS (control) at 22 °C for 2 h. After extensive washing, the bacterial cells were added to PBLs (1:1 ratio) and incubated for 1 h. The cells were extensively washed to remove non-infecting bacteria. PBLs were then lysed with 1% Triton X-100, and the cell lysate was plated in LB agar plates. The bacterial colonies were counted and verified as previously reported [38]. To examine the effects of HMGB1/HMG20A on bacteria-induced ROS production, PBLs were pre-incubated with DCFH-DA and seeded in 96-well plates as described above. *P. fluorescens* (1 × 10^6^) was added to PBLs (1 × 10^6^) containing HMGB1, HMG20A, or Trx at a final concentration of 50 μg/mL or PBS (control) followed by incubation for 2, 3, or 4 h, and ROS production was then measured as described above.

### 2.11. Statistical Analysis

All of the assays were performed in triplicate or quadruplicate. SPSS version 17.0 software (SPSS Inc., Chicago, IL, USA) was used for Student’s *t*-test statistical analysis, and statistical significance was defined as *p* < 0.05.

## 3. Results

### 3.1. Sequence Characteristics of Flounder HMGB1 and HMG20A

**Figure 1:**
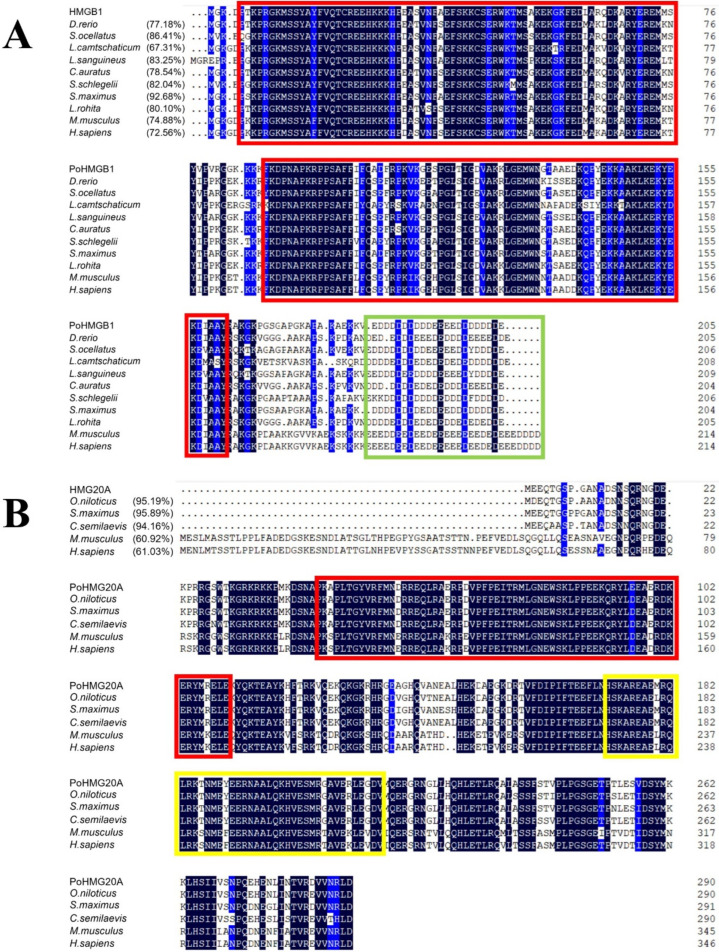
Alignment of the protein sequences of HMGB1 and HMG20A homologues. Flounder HMGB1 (**A**) and HMG20A (**B**) were aligned with homologues from other species. Overall protein sequence identities are indicated on the left of sequences. The identical residues are indicated in black; the residues that are ≥75% identical are indicated in blue; the red boxes indicate the HMG domains; the green box in (**A**) indicates the acidic tail; and the yellow box in (**B**) indicates the coiled coil region. HMGB1 GenBank accession numbers are as follows: *Paralichthys olivaceus*, AGT28457.1; *Danio rerio*, NP_955849.2; *Sciaenops ocellatus*, ADX06860.1; *Lethenteron camtschaticum*, AEH59759.1; *Lutjanus sanguineus*, AIK66523.1; *Carassius auratus*, AHI15743.1; *Sebastes schlegelii*, AMQ67246.1; *Scophthalmus maximus*, AXB88328.1; *Labeo rohita*, ARX80203.1; *Homo sapiens*, NP_002119.1; *Mus musculus*, NP_034569.1. HMG20A GenBank accession numbers are as follows: *Paralichthys olivaceus*, XP_019969411.1; *Oreochromis niloticus*, XP_005453160.1; *Scophthalmus maximus*, XP_035498689.2; *Cynoglossus semilaevis*, XP_016888886.1; *Mus musculus*, AAH13804.1; *Homo sapiens*, XP_047288065.1.

HMGB1 comprised 205 amino acid residues with two HMG boxes (residues 5–77 and 94–161) and an acidic motif of 22 D/E residues (Figure 1A). The calculated molecular mass of HMGB1 is 23.25 kDa. The theoretical pI of HMGB1 is 7.34. HMGB1 shares 67.31–92.68% overall protein sequence identity with the teleost HMGB1 from *Danio rerio*, *Sciaenops ocellatus*, *Lethenteron camtschaticum*, *Lutjanus sanguineus*, *Carassius auratus*, *Sebastes schlegelii*, *Scophthalmus maximus*, and *Labeo rohita*. The sequence identity between flounder HMGB1 and human HMGB1 is 72.56%. HMG20A comprised 291 residues with an HMG box (residues 45–110) and a coiled coil region (residues 172–215) (Figure 1B). The HMG20A molecular mass was calculated to be 33.91 kDa with a theoretical pI of 9.74. HMG20A shares 94.16–95.89% overall protein sequence identity with the HMG20A of *Oreochromis niloticus*, *Scophthalmus maximus*, and *Cynoglossus semilaevis*. The sequence identity between flounder HMG20A and human HMG20A is 61.03%.

### 3.2. HMGB1 Is Released from PBLs during Bacterial Infection and Induces PBL Activation

**Figure 2:**
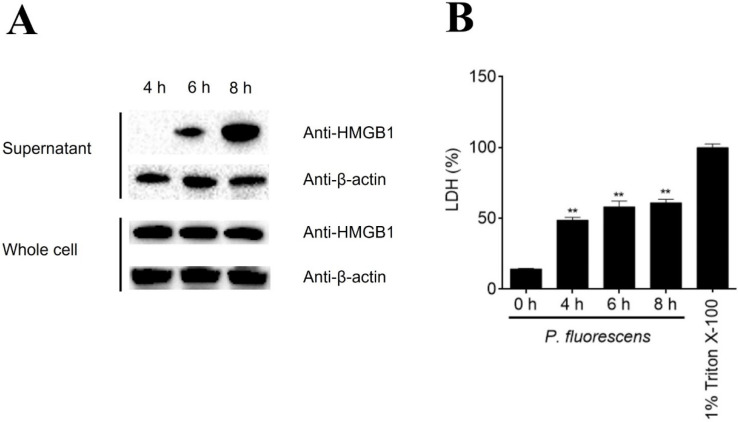
Detection of HMGB1 and LDH release from peripheral blood leukocytes during bacterial infection. (**A**) Flounder peripheral blood leukocytes (PBLs) were infected with *Pseudomonas fluorescens*. At 4, 6, and 8 h postinfection (hpi), HMGB1 in the whole cells and the culture supernatant were detected by immunoblotting. (**B**) PBLs were infected as above. At 0, 4, 6, and 8 hpi, LDH in the supernatant and whole cells was determined. PBLs lysed by 1% Triton X-100 were used as the maximum of LDH release. Values of triplicate experiments are shown as means ± SD. ** *p* < 0.01.

**Figure 3:**
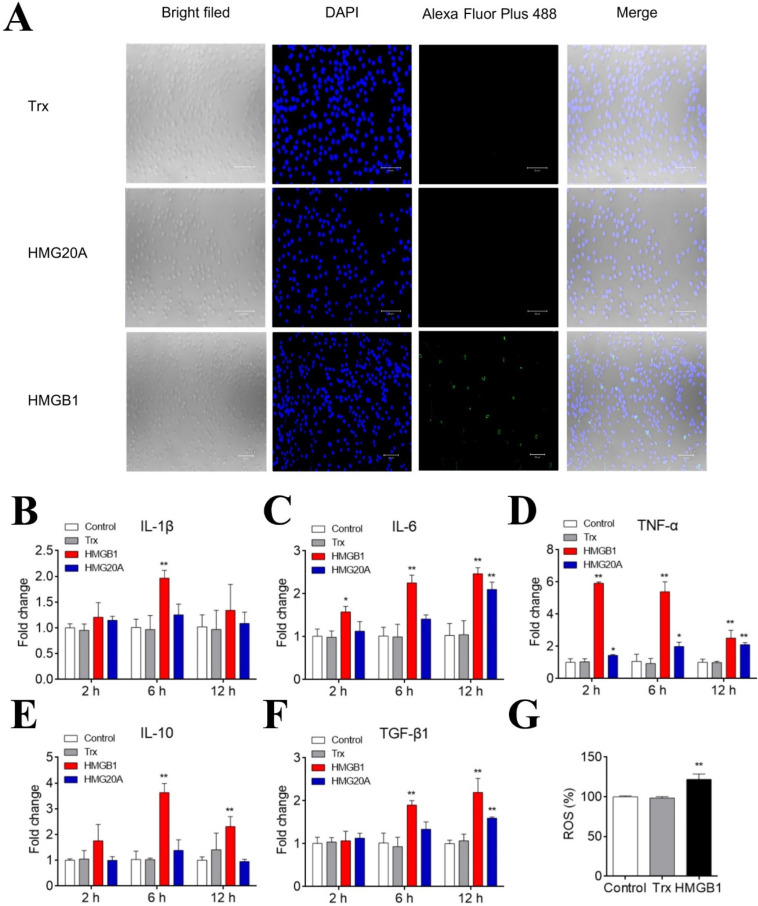
HMGB1 binds to and activates PBLs. (**A**) Flounder PBLs were isolated and incubated with HMGB1, HMG20A, or Trx for 2 h. The bound proteins were indicated by anti-His tag antibody and Alexa Fluor Plus 488 labeled secondary antibody. DAPI was used to indicate nuclei. The binding of HMGB1 to PBLs was observed using a confocal microscope. Bar size, 20 μm. (**B**–**F**) PBLs were treated with or without (control) HMGB1, HMG20A, or Trx. IL-1β (**B**), IL-6 (**C**), TNF-α (**D**), IL-10 (**E**), and TGF-β1 (**F**) expression were determined at different hours by qRT-PCR. (**G**) PBLs were treated with HMGB1 as above for 2 h, and ROS production was determined. For panels (**B**–**G**), values of four replicate experiments are shown as means ± SD. ** *p* < 0.01; * *p* < 0.05.

The HMGB1 and HMG20A expression levels in nine tissues were detected by qRT-PCR. The highest expression levels of HMGB1 and HMG20A were detected in the brain and intestine, respectively, while the lowest expression levels of HMGB1 and HMG20A were detected in the gill and muscle, respectively (Figure S2). Immunoblotting showed that when flounder PBLs were infected with *P. fluorescence* for 6 and 8 h, HMGB1 was released in the culture supernatant in a time-dependent manner (Figure 2A). On the same time course, LDH and β-actin releases were also detected (Figure 2B). To examine whether extracellular HMGB1 could interact with PBLs, the cells were incubated with recombinant HMGB1. Subsequent microscopy and qRT-PCR analyses showed that HMGB1, but not HMG20A, bound apparently to PBLs (Figure 3A). HMGB1 induced time-dependent expression of cytokines and ROS production in PBLs (Figure 3B–G). Specifically, IL-6 and TNF-α expression levels were significantly increased at 2 to 12 hpi; IL-1β expression was significantly increased at 6 hpi, while IL-10 and TGF-β1 expression levels were increased significantly at 6 and 12 hpi. In contrast, HMG20A had no significant effect on IL-1β and IL-10 expression and induced much weaker expression of other cytokines compared to HMGB1.

### 3.3. HMGB1 Binds to Various Bacteria in a HMG20A-Competitive Manner and Causes Bacterial Agglutination

**Figure 4:**
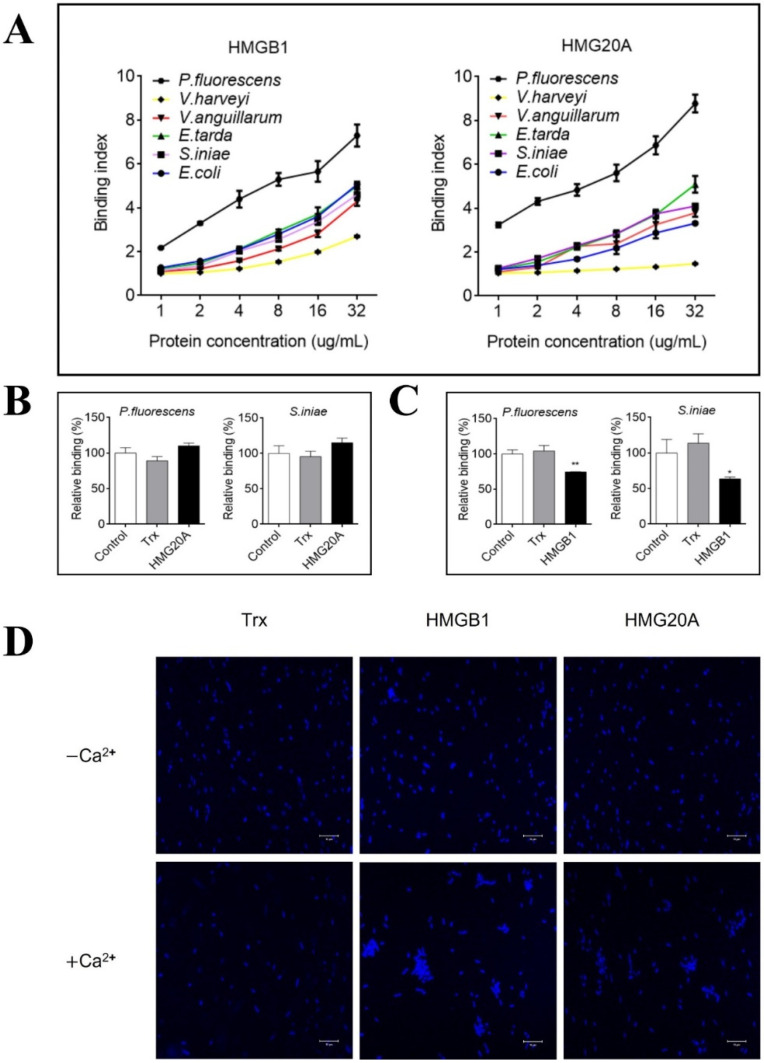
Interaction of HMGB1 and HMG20A with bacteria. (**A**) HMGB1 and HMG20A were incubated with *Pseudomonas fluorescens*, *Vibrio harveyi*, *Vibrio anguillarum*, *Streptococcus iniae*, *Edwardsiella tarda*, or *Escherichia coli* at different concentrations for 2 h. The binding of HMGB1 and HMG20A to bacteria was determined by ELISA. (**B**) *P. fluorescens* and *S.* *iniae* were pre-incubated with PBS (control), Trx, or HMG20A and then incubated with HMGB1. Bacteria-bound HMGB1 protein was determined as above. (**C**) *P. fluorescens* and *S.* *iniae* were pre-incubated with PBS (control), Trx, or HMGB1 and then incubated with HMG20A. Bacteria-bound HMG20A protein was determined as above. For panels (**B**,**C**), values of triplicate experiments are shown as means ± SD. ** *p* < 0.01; * *p* < 0.05. (**D**) *P. fluorescens* was incubated with HMGB1, HMG20A, or Trx for 2 h in the presence or absence of calcium. DAPI staining was performed to indicate bacterial cells. The bacterial-binding proteins were observed with a confocal microscope. Bar size, 10 μm.

Since bacterial infection induced HMGB1 release, we examined whether HMGB1 could directly interact with bacteria. The results showed that HMGB1 bound to a number of bacteria, in particular *P. fluorescence*, in a dose-dependent manner (Figure 4A). Similar binding patterns were observed with HMG20A (Figure 4A). Microscopic observation verified the binding of HMGB1 and HMG20A to bacterial cells (Figure S3). Since HMGB1 and HMG20A exhibited comparable bacterial binding profiles, we examined whether there was binding competition between the two proteins. We found that for both the Gram-negative *P. fluorescence* and the Gram-positive *S. iniae*, pre-incubation of the bacteria with HMG20A had no effect on subsequent HMGB1 binding (Figure 4B), whereas pre-incubation of the bacteria with HMGB1 significantly reduced subsequent HMG20A binding (Figure 4C). Microscopy revealed agglutination of HMGB1-bound bacteria (Figure 4D). HMG20A could also agglutinate bacterial cells but to a lesser degree compared to HMGB1. Moreover, the agglutinating effects of both HMGB1 and HMG20A required Ca^2+^ (Figure 4D).

### 3.4. HMGB1 Promotes the Antibacterial Activity of PBLs and Impairs Bacterial Infection

**Figure 5:**
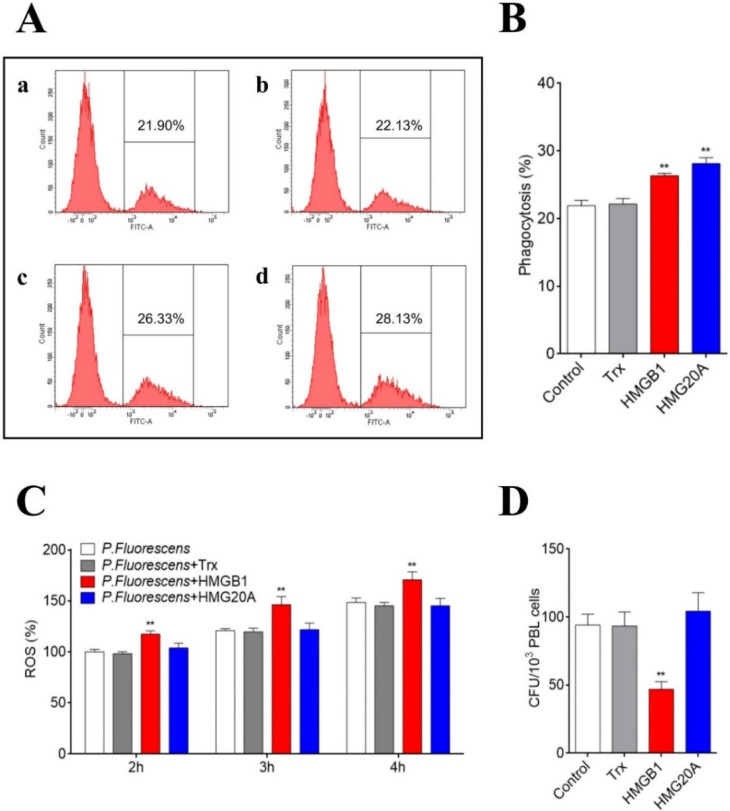
Effects of HMGB1 on bacterial infection. (**A**,**B**) Flounder PBLs were infected with FITC-labeled *Pseudomonas fluorescens* that were pre-incubated with PBS (control, A(a)), HMGB1 (A(c)), HMG20A (A(d)), or Trx (A(b)). Phagocytosis of bacteria was determined by flow cytometry (**A**). The phagocytic percentages are shown in (**B**). (**C**) PBLs were infected with *P. fluorescens* in the presence or absence (control) of HMGB1, HMG20A, or Trx. ROS production was measured at 2, 3, and 4 h postinfection. (**D**) *P. fluorescens* was pre-incubated with or without (control) HMGB1, HMG20A, or Trx, followed by incubation with PBLs. The number of PBL-infected bacteria was determined by counting bacterial colonies. For panels B to D, values of triplicate/quadruplicate experiments are shown as means ± SD. ** *p* < 0.01.

Since HMGB1/HMG20A interacted with both PBLs and bacterial pathogens, we investigated whether these proteins could affect the bacterial infection of PBLs. An FACS analysis showed that HMGB1 and HMG20A treatment significantly increased bacterial phagocytosis by PBLs (Figure 5A,B). HMGB1, but not HMG20A, also significantly increased the ROS level of bacteria-infected cells (Figure 5C). Consistently, when PBLs were treated with *P. fluorescence*, the presence of HMGB1 significantly decreased the number of live bacteria from the infected cells (Figure 5D). In contrast, the treatment with HMG20A had little effect on bacterial infection.

## 4. Discussion

To date, HMGB1 has been characterized in a number of species, and its amino acid sequence has been shown to be highly conserved during evolution [6]. The classical HMG boxes (box A and box B) and the C-terminal acidic motif are universally present in mammalian and teleost HMGB1 [8]. In mammals, the HMG boxes are responsible for DNA binding, while the acidic tail regulates the DNA-binding activity of HMG boxes [39]. In this study, we found that HMGB1 from flounder shared high sequence identity with those from other fish, all of which possess the conserved box A/B and acidic terminus, suggesting a functional conservation of HMGB1 in higher and lower vertebrates. In mammals, HMGB1 is expressed in nearly all tissues, especially the lymphoid tissues and liver [40]. In teleosts, different expression patterns of HMGB1 have been reported. For example, in goldfish, black rockfish, and turbot, HMGB1 is expressed at the highest and lowest levels in the brain and liver, respectively [26]. In rohu, the highest HMGB1 expression was detected in the blood [40], while in red drum, the highest and lowest expression of HMGB1 occurred in the muscle and kidney, respectively [28]. In the present study, flounder HMGB1 was expressed at much higher levels in the brain and intestine than that in the gill or muscle. For HMG20A, its expression in Nile tilapia was highest in the liver and lowest in the gill [29]. In flounder, we found that the expression level of HMG20A was highest in the intestine while being expressed at the lowest level in the muscle. The diverse patterns of HMGB expression suggest that the HMGB members may be regulated by different mechanisms in various fish species.

In mammals, in addition to functioning as a nuclear protein, released HMGB1 can act as a signal molecule to regulate cellular activity [41]. The extracellular production of HMGB1 can occur via active secretion or passive release [7]. The release of HMGB1 occurs during different types of regulated cell death, such as pyroptosis, necroptosis, necrosis, and autophagy [42]. Under these conditions, the integrity of the cell membrane is interrupted so that HMGB1 can be liberated into the extracellular milieu. Previous reports showed that human/mouse HMGB1 was secreted by monocytes during lipopolysaccharide (LPS) treatment and released by neural cells and dendritic cells during various stresses [43,44]. HMGB1 was also discharged by macrophages under stresses caused by bacterial and viral pathogens [45,46]. In teleosts, the HMGB1 of red drum was reported to be released from macrophages treated with *Edwardsiella tarda* [28]. HMGB1 expression was enhanced in humphead snapper and grass carp during the infection of *Vibrio harveyi* and grass carp reovirus, respectively [25,47]. In invertebrates, HMGB1 was involved in the immune response to pathogen-associated molecular patterns (PAMP). For example, in Pacific oyster, *Crassostrea gigas*, LPS induced HMGB1 translocation from the cell nucleus to the cytoplasm [48]; in scallop, *Chlamys farreri*, HMGB1 expression was significantly upregulated in response to LPS, peptidoglycan, and glucan stimulation [49]. In flounder, our study showed that HMGB1 was produced extracellularly from PBLs after *P. fluorescens* challenge. The correlation in time course between HMGB1 and LDH release indicated that the observed HMGB1 release was probably a passive process resulting from cell death/injury caused by *P. fluorescens* infection.

Released HMGB1 was reported to engage a variety of cellular membrane receptors, such as TLR2, TLR4, and TLR9, and to exert a stimulatory effect on various types of cells by activating the MAPK and NF-κB pathways, resulting in inflammation [7,8,50,51,52]. For example, human HMGB1 can form a complex with CXCL12 to bind to CXCR4 receptors, thereby promoting the proinflammatory immune response [53]; the 89–108 residues of HMGB1 can interact with TLR4 and stimulate pro-inflammatory signals [17], and the 150–183 residues of HMGB1 can bind to RAGE to increase cell migration signals [16]. In some fish species, HMGB1 can also function as a pro-inflammatory factor. Humphead snapper HMGB1 was reported to increase IL-6 expression in head kidney leukocytes [47]. Goldfish HMGB1 enhanced the expression of TNF-α and IL-1β in macrophages [26]. In black rockfish, HMGB1 promoted the expression of TNF-α, TNF13B, and IL-1β [54]. In pufferfish, HMGB1 was able to synergize with CpG-oligodeoxynucleotide to enhance the expression of IL-1β, IL-6, TNF-α, and IFN-γ [55]. In the present study, we found that flounder HMGB1 stimulated the expression of both pro-inflammatory cytokines (e.g., IL-6 and TNF-α) and anti-inflammatory cytokines (e.g., IL-10 and TGF-β1) at the early and late stages, respectively. The dynamic expression patterns of these cytokines implied a tight control of HMGB1-mediated inflammation in flounder. In addition to the inflammatory response, flounder HMGB1 also promoted ROS production, similar to that observed with human HMGB1 [56].

Previous studies showed that mammalian HMGB1 could bind various pathogenic molecules, including LPS, lipoteichoic acid (LTA), bacterial DNA, and virus-derived nucleic acids, and amplify the immune responses induced by these PAMPs [7,57,58,59]. In fish, one study showed that HMGB1 from turbot bound LPS, peptidoglycan, and LTA [60]. To date, no direct interaction between HMGB1 and bacterial cells has been documented. Compared to HMGB1, much less is known about HMG20A, and it is unclear whether HMG20A can interact with PAMPs. In this study, we found that flounder HMGB1 as well as HMG20A could bind to various bacteria. It is interesting that the HMGB1–bacteria interaction blocked subsequent HMG20A interaction with bacteria, while the HMG20A–bacteria interaction did not affect subsequent binding of HMGB1 to bacteria. These results suggest that HMGB1 and HMG20A probably interacted with the same receptor molecule on bacterial cells, but the binding affinity of HMGB1 was likely much stronger than that of HMG20A.

The process of phagocytosis is critical for the host to remove bacterial pathogens. In mammals, HMGB1 is known to facilitate the phagocytosis of RAW264.7 cells [61]. Herein, the phagocytosis of flounder PBLs was increased significantly by HMGB1, which might be due to the ability of HMGB1 to cause bacterial agglutination. In addition to phagocytosis, HMGB1 also enhanced the ROS production in bacteria-infected PBLs. Consistently, the presence of HMGB1 reduced the infection of *P. fluorescens* to PBLs, most likely as a result of an augmented inflammatory response, phagocytosis, and ROS killing elicited by HMGB1.

In conclusion, our results indicate an important role of flounder HMGB1 in antibacterial immunity. HMGB1 is a pathogen-induced DAMP that not only activates fish immune cells but also binds directly to bacteria, thereby promoting bacterial clearance. These findings add new insights into the function of fish HMGB proteins.

## Data Availability

The data presented in this study are available in the article or Supplementary Materials.

## Supplementary Materials

The following supporting information can be downloaded at: https://www.mdpi.com/article/10.3390/genes13091509/s1, Figure S1. Analysis of HMGB1 (A), HMG20A (B), and Trx (C) used in this study. Figure S2. In vivo expression of HMGB1 and HMG20A in flounder. Figure S3. Microscopic observation of HMGB1/HMG20A binding to bacterial cells. Table S1. List of primers used in this study.
