# Supplementary material for: Japanese Flounder HMGB1: A DAMP Molecule That Promotes Antimicrobial Immunity by Interacting with Immune Cells and Bacterial Pathogen
Source: Genes (Basel). 2022 Aug 23;13(9):1509. doi: 10.3390/genes13091509 (PMC9498587; doi:10.3390/genes13091509)
Supplement: Supplementary file 1 [file genes-13-01509-s001.zip › genes-1867374-suppl.pdf]

Supplemental data

Table S1. List of primers used in this study.

Primer	Sequences (5′→3′) [a]	Target gene
HMGB1-F	GTGCCGCGCGGCAGCCATATGGGGAAGGATCCAACCA	HMGB1
HMGB1-R	ACGGAGCTCGAATTCGGATCCCTACTCGTCATCATCATCATCGTCGTCTTCCTC	HMGB1
HMG20A-F	GTGCCGCGCGGCAGCCATATGGAGGAGCAGACAGGCT	HMG20A
HMG20A-R	ACGGAGCTCGAATTCGGATCCTTACCTGTCCAAACGGTT	HMG20A
RT-HMGB1-F	GAAGGATCCAACCAAGCCGA	HMGB1
RT-HMGB1-R	TCGCTCAGAGCACTTCTTGG	HMGB1
RT-HMG20A-F	CATCCCGATCTTCACCGAGG	HMG20A
RT-HMG20A-R	CGTTACGCTCCTCGTACTCC	HMG20A
β-actin-F	AACCGCTGCCTCCTCCTCAT	β-actin
β-actin-R	TCGGGACAACGGAACCTCTC	β-actin
18S rRNA-F	GGTCTGTGATGCCCTTAGATGTC	18S rRNA
18S rRNA-R	AGTGGGGTTCAGCGGGTTAC	18S rRNA
TNF-α-F	CCACAACACACTGAGGCAAA	TNF-α
TNF-α-R	TCCACACCAGCTTGTTTTCG	TNF-α
IL-6-F	TAACCGCTCACCACCAGAAA	IL-6
IL-6-R	AGACAAGCCTCCTTGCTGAA	IL-6
IL-1β-F	CAGCACATCAGAGCAAGACAACA	IL-1β
IL-1β-R	TGGTAGCACCGGGCATTCT	IL-1β
TGF-β1-F	TGTTGACTAGTGCGGAGCTG	TGF-β1
TGF-β1-R	GTTGGAAACAACGCTCGTCC	TGF-β1
IL-10-F	GCCTCACGTGGAGTCCATAC	IL-10
IL-10-R	CAGCTCGCCCATGGCTTTAT	IL-10

[a]Underlined nucleotides are restriction sites.

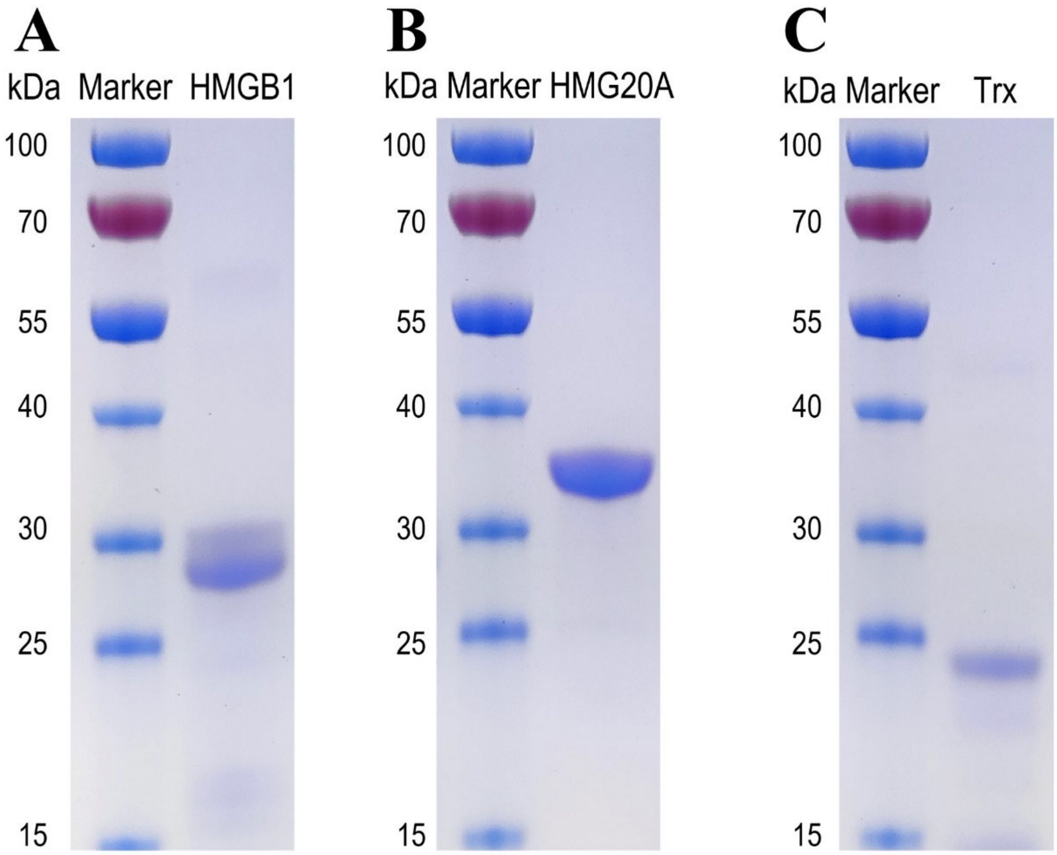

Figure S1. Analysis of HMGB1 (A), HMG20A (B), and Trx (C) used in this study. Recombinant proteins were purified and analyzed by SDS-PAGE and CBB R-250 staining.

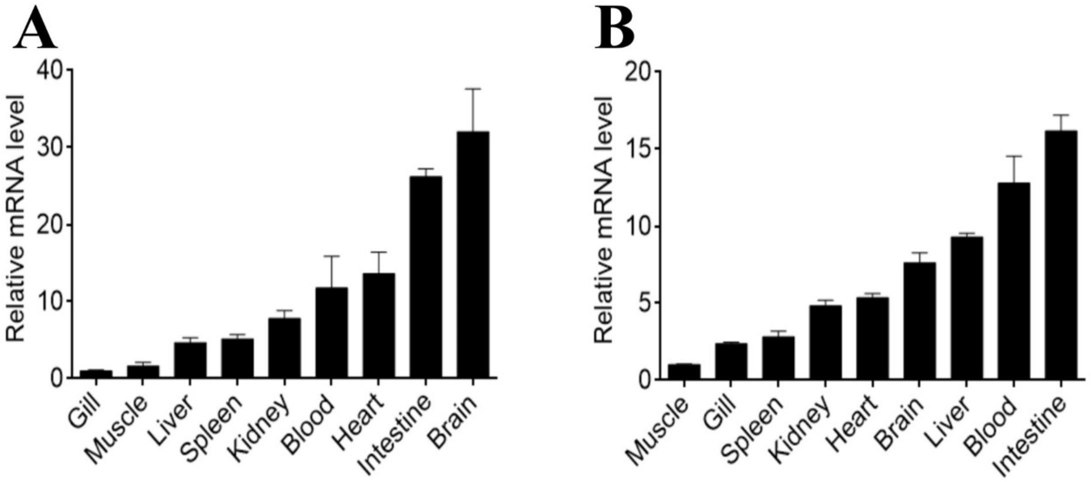

Figure S2. ***In vivo*** **expression of HMGB1 and HMG20A in flounder.** HMGB1 (A) and HMG20A (B) expression in nine tissues of flounder was determined by qRT-PCR. The expression level in gill (A) or muscle (B) was set as 1. Data from three independent assays are shown as means ± SEM.

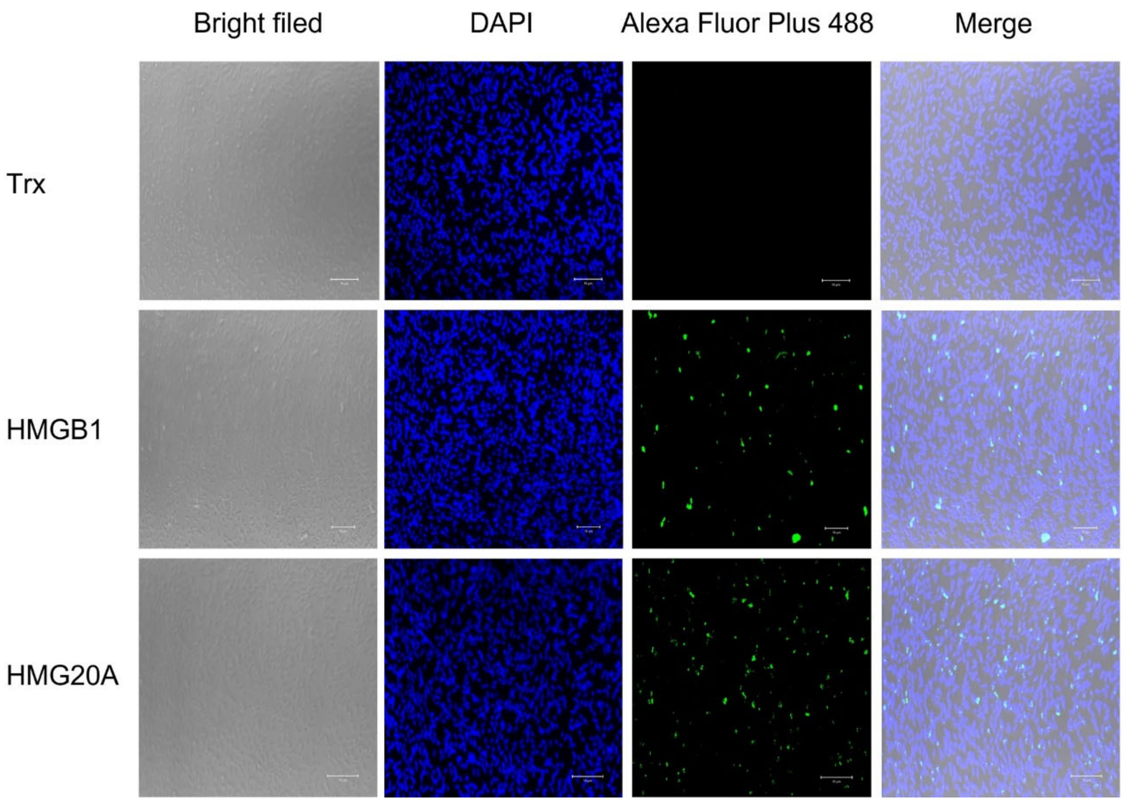

Figure S3. Microscopic observation of HMGB1/HMG20A binding to bacterial cells. *P. fluorescens* was incubated with HMGB1, HMG20A or Trx, and further treated with anti-His tag antibody and Alexa Fluor Plus 488-labeled secondary antibody. DAPI staining was performed to indicate cells. Images were taken by a fluorescence microscope. Bar size, 10 μm.
